# Knowledge, attitude, and self-care practice regarding glaucoma among hospital workers at a tertiary center in South-South Nigeria

**DOI:** 10.1186/s12886-026-04832-7

**Published:** 2026-04-17

**Authors:** Thelma Imaobong Ndife

**Affiliations:** Department of Ophthalmology, University of Uyo Teaching Hospital, Uyo, Akwa Ibom State Nigeria

**Keywords:** Glaucoma, Knowledge, Attitude, Self-care practice, Hospital workers, South-South Nigeria

## Abstract

**Background:**

Undetected glaucoma among hospital workers poses a significant risk of preventable vision loss within the healthcare workforce, potentially impacting the delivery of care. The purpose of this study was to assess the medical and non-medical hospital workers’ knowledge, attitude and self-care practice regarding glaucoma.

**Methods:**

In a hospital-based cross-sectional study, a stratified random sample of 401 hospital workers, including 206 (51.4%) medical and 195 (48.6%) non-medical personnel, were administered a structured knowledge, attitude, and practice (KAP) questionnaire on glaucoma. Differences in glaucoma knowledge among staff cadres were compared using the Chi-square test. The predictors of KAP were analysed using multivariate logistic regression.

**Results:**

Glaucoma was defined by respondents as blindness (26.9%), raised intraocular pressure (24.7%), and optic nerve damage (9.5%). Common risk factors identified were hypertension (87.9%), diabetes (86%) and positive family history of glaucoma (80.8%). Only 8.0% believed that early diagnosis and treatment could prevent blindness. Strikingly, 70.1% of hospital workers had never undergone an eye examination. Workers (68.6%) would rather use medications than accept surgery, adding adjunctive modalities like prayers and traditional medicines were reported by 58.9% of the workers. Medical personnel demonstrated greater knowledge of glaucoma (*p* < .001) and were more likely to accept surgical intervention (*p* = .003). Good knowledge of glaucoma, positive attitude and good self-care practice were reported by 2.5%, 10.7% and 14.4% of the workers. Secondary level educational was predictive of positive attitude (OR 3.35, 95% CI 1.60-7.03, *P*= .001), non-medical cadre was predictive of poor glaucoma practice (OR 0.22. 95% CI 0.10-0.48, *p*<.001) while a visit to the ophthalmologist was predictive of good glaucoma practice (OR 19.92, 95% CI 8.78-42.94, *p*<.001).

**Conclusion:**

All hospital personnel should be re-educated about the potential blinding effect of glaucoma. The need for timely, regular, comprehensive glaucoma evaluation and the adoption of early, appropriate management is advocated.

**Supplementary Information:**

The online version contains supplementary material available at 10.1186/s12886-026-04832-7.

## Introduction

Glaucoma affects 3.54% of individuals aged 40 to 80 years globally [1]. In 2013, approximately 64.3 million people in this age group were affected, with projections increasing to 76.0 million in 2020 and 111.8 million by 2040 [1]. As populations age, the prevalence of glaucoma is anticipated to rise further [2]. In Africa, the prevalence is higher at 5.59%, with primary open-angle glaucoma (POAG) particularly common in West Africa [3]. Glaucoma presents a major public health challenge in Africa for several reasons. POAG is frequently asymptomatic in its early stages, resulting in about one-third of patients being blind at the time of seeking care [4]. Myths and misconceptions about glaucoma further impede early diagnosis and timely treatment [5]. Surgical intervention is rarely chosen by patients [6], and the cost of medication remains prohibitive for many African individuals [7]. With this scenario, it is imperative that hospital workers possess adequate knowledge of glaucoma.

Hospitals serve a vital function in delivering clear and accurate information about glaucoma to patients and communities. Since patients often seek guidance from a range of hospital staff, it is essential that all personnel, not solely eye health workers, possess comprehensive knowledge and demonstrate a supportive approach to glaucoma care. Knowledgeable workers can identify the risk factors for glaucoma; encourage screening and patient compliance with eye drop routine. Workers themselves are not immune to the disease. Significant proportions are aged above 40 years; a risk factor which when coupled with the presence of systemic comorbidities makes them quite vulnerable to glaucoma. A thorough understanding of glaucoma not only encompasses factual knowledge but also shapes staff attitude and behaviors, both of which are crucial for fostering effective self-care [8].

Studies in Nigeria have revealed that many healthcare workers lack adequate knowledge about glaucoma and frequently neglect their own eye health [9–11]. Assessing the hospital workers’ perception in areas with high glaucoma prevalence is critical for effective intervention planning. A cross-sectional survey was conducted using structured questionnaires administered to a sample of hospital staff. Investigating KAP at a tertiary hospital in South-South Nigeria, the study intends to generate insights to inform educational initiatives and strategic planning.

## Methodology

**Study Design and target population**: An observational cross-sectional study design was adopted. The study was carried out at University of Uyo Teaching Hospital, a tertiary care setup. Staff members were categorized into two cadres: Medical and non-medical. The medical cadre comprised of doctors, nurses, physiotherapists, laboratory scientists and pharmacists while the non-medical cadre was made of administrative personnel, health information managers, information technology and maintenance crew, accountants, auditors, security and transport officers.

**Sample Size Calculation**: The sample size (n) was determined by Cochran’s sample size formula with the assumption of 95% confidence level (z = 1.96), e the margin of error is 5%, p is the (estimated) proportion of the population which has the attribute in question which equals 50% (or 0.5), and q is 1 – p: n = z^2^pq /e^2^. The required minimum sample size (n) for this study was 385. 10% was added to account for non-response = 424.

Stratified random sampling was employed. From about 1,500 staff strength, the computer randomly picked 212 names from each cadre specific hospital register. After signing informed consent, 206 medical and 195 non-medical staff filled the questionnaire. Thus, 401 personnel comprising 26.7% of the target population were interviewed.

**Inclusion Criteria**: Hospital personnel who were above 18 years of age and willing to participate in the study.

**Exclusion Criteria**: Personnel from the ophthalmology unit were excluded.

### Operational definitions

Knowledge: The understanding of different aspects of glaucoma like its definition, presentation, risk factors and treatment options.

Attitude: The health workers’ mindset towards glaucoma knowledge and management measured with questions about swift compliance with prescribed treatment protocol, strict adherence to dose/ instillation time of prescribed medications and regular keeping of the ophthalmologists’ appointment.

Self- care practice: A collection of activities that an individual with glaucoma would perform on their own to control and maintain vision. These include undergoing a comprehensive glaucoma evaluation and intra-ocular pressure check, promptly accepting glaucoma surgery when recommended, compliance with a change of treatment plan, avoiding the use of adjunctive modalities not endorsed by the doctor.

**Data Collection and Coding**: Trained interviewers assessed each of the personnel about their knowledge, attitude and self-care practice in face-to-face interviews using a structured questionnaire.

The questionnaire consisted of three sections which had been modified from a similar previous research questionnaire [12] to enhance clarity and relevance. The questionnaire (Appendix 1) was reviewed for content validity, and it was piloted with hospital staff. A Cronbach’s alpha of 0.86 showed internal consistency. Analysis of the study data did not include information obtained from the pilot study.

Section A: Comprised questions regarding socio-demographic characteristics (age, gender, marital status, educational level, and cadre) of staff. Personnel were also asked about personal or family history of glaucoma.

Section B: Focused on knowledge about glaucoma. It consisted of 20 questions about the definition, risk factors, symptoms, possibility of treatment and the available treatment options for glaucoma. Each had 3 possible responses; Yes, No and Can’t Say. A correct answer was awarded a score of 1 point while the wrong answer and Can’t Say were scored 0. A maximum of 20 points were allotted to Section B. Knowledge scores were graded as: Poor 0–14 = 0 < 75% and Good 15–20 = ≥ 75%.

Section C: Focused on attitude and self-care practice related to glaucoma. In the event of a glaucoma diagnosis, their attitude towards the use of topical medications as recommended, judicious adherence to doctors’ appointments, and the preferred treatment modality between medication and surgery were confirmed. The last time a visit was paid to an ophthalmologist was queried. Three questions were focused on the personnel’s practice. If the only treatment option available was surgery, workers were asked if they would promptly accept surgery, defer surgery but continue with medications or add adjunctive medications. A visit to the ophthalmologist was awarded weighted scores as follows; < 1 year = 5, 1–2 years = 4, ≥ 3 years = 3, Can’t Remember = 2, Never = 1. Other questions were awarded a 5 point Linkert score: Strongly Disagree = 1, Disagree = 2, Neutral = 3, Agree = 4 and Strongly Agree = 5 points. Attitude was scored maximum of 15points and graded: Negative 0–11 = 0 < 75%, Positive 12–15 = ≥ 75%. Self-care practice was scored maximum of 20 points and graded: Poor 0–14 = 0 < 75%, Good 15–20 = ≥ 75%.

A composite maximum score of 55 was summed up for KAP related responses. Scores were graded as: Poor, 0–41 = 0 < 75%, Good, 42–55 = ≥ 75%.

**Data Analysis**: Data was analyzed using IBM SPSS Statistics version 29, IBM Corp. (Armonk, NY). Data was summarized as averages, proportions and percentages. Descriptive statistics were reported using tables. Categorical variables were compared using the chi-square test. Knowledge, attitude and practice grades was taken as the dependent variable while gender, age, marital status, educational level, cadre of staff, last visit to an ophthalmologist, personal and family history of glaucoma were the independent variables. The association between the dependent and independent variables was tested with Chi-square test while the predictors of KAP were analysed using Multivariate Logistic Regression. A two-tailed p-value of less than 0.05 was considered statistically significant at 95% confidence interval.

## Results

### Socio-demographic characteristics:

The distribution of medical and non-medical personnel by their socio-demographic characteristics and glaucoma history is presented on Table 1. A total of 401 responses were analyzed comprising 206 (51.4%) medical and 195 (48.6%) non-medical workers respectively. The differences in age, gender, marital status and educational level between medical and non-medical personnel were not statistically significant. The average age of responders was 41.32 ± 10.06 years with age range of 18 to 79 years. The sample included a higher proportion of female (75.8%) compared to male personnel (24.2%), with a male-to-female ratio of 1:3. Few personnel 11 (2.7%) had a personal diagnosis of glaucoma and 43 (10.7%) had a positive family history of glaucoma.

**Table 1:** Socio-demographic characteristics and glaucoma history of medical and non-medical hospital personnel

Socio-demographic characteristic		Total frequency *n* = 401 (100%)	Medical *n* = 206 (51.4%)	Non-medical *n* = 195 (48.6%)	*p* value
Age group (years)	18–30	66 (16.5)	36(9.0)	30 (7.5)	0.365
	31–50	267 (66.6)	132(32.9)	135 (33.7)	
	51–70	67 (16.7)	38 (9.5)	29 (7.2)	
	> 71	1 (0.2)	0 (0)	1 (0.2)	
Mean Age (years)		41.32 ± 10.06	41.35 ± 10.09	41.27 ± 10.05	
Gender	Female	304 (75.8)	163(40.6)	141(35.2)	0.098
	Male	97 (24.2)	43(10.7)	54 (13.5)	
Marital Status	Married	279 (69.6)	142(35.4)	137 (34.2)	0.584
	Single	108 (26.9)	55(13.7)	53 (13.2)	
	Widowed	14 (3.5)	9(2.2)	5(1.3)	
Educational level	Primary	5 (1.2)	3(0.7)	2(0.5)	0.926
	Secondary	57(14.2)	29(7.2)	28(7.0)	
	Tertiary	339 (84.5)	174(43.4)	165(41.1)	
History of glaucoma	Personal	11(2.7)	7(1.7)	4(1.0)	0.489
	Family	43( 10.7)	31(7.7)	12(3.0)	< 0.005*

Key: * =Significant P- value

### Knowledge of glaucoma

The responses regarding knowledge of glaucoma and the perceived treatment options are detailed in Table 2. Some personnel, 108 (26.9%) defined glaucoma as blindness, and 75 (18.6%) specifically linked it to irreversible blindness. Additionally, 99 (24.7%) personnel defined glaucoma with raised intraocular pressure, while 38 (9.5%) recognized it as being related to optic nerve damage. Some responders, 50 (12.5%) were able to associate glaucoma with more than one correct description. Only 4.5% of responders described glaucoma as asymptomatic, and 15.5% noted that early glaucoma is painless. A mere 4.0% understood that peripheral visual field is often lost in the early stages of the disease. While 67.6% believed that glaucoma could be treated, only 8% thought that early diagnosis and treatment could prevent blindness. More than a half of the workers reported they were aware of medications 254 (63.3%) and surgery 236 (58.8%) as treatment options for glaucoma. Medical professionals reported a greater family history of glaucoma (*p* < .005), demonstrated significantly better understanding in describing glaucoma (*p* < .001) and better knowledge about the available treatment options (*p* = .028).

**Table 2:** Knowledge of glaucoma among medical and non-medical personnel

Question	Associations	Yes *n* = 401, (%)	Medical professional *n* = 206, (%)	Non-medical professional *n* = 195, (%)	*p* value
How would you describe glaucoma	Raised Intraocular pressure	99(24.7)	41(10.2)	58(14.5)	< 0.001*
	Optic nerve damage	38(9.5)	31(7.7)	7(1.7)	
	Causes blindness	108(26.9)	43(10.7)	65(16.2)	
	Blindness is irreversible	75 (18.6)	40(9.9)	35(8.7)	
How does glaucoma present	Early glaucoma is painless	62(15.5)	28(7.0)	34(8.5)	0.201
	Glaucoma is asymptomatic	18(4.5)	11(2.7)	7(1.7)	
	Glaucoma Affects side vision before central vision	16(4.0)	8(2.0)	8(2.0)	0.696
Treatment	Treatment of Glaucoma is possible	279(67.6)	126(31.4)	153(38.2)	0.085
	Blindness from Glaucoma can be prevented by early diagnosis and treatment	32(8.0)	20(5.0)	12(3.0)	0.373
What are the treatment options for Glaucoma	Medication/Eye drop	254(63.3)	122(30.4)	132(32.9)	0.028*
	Surgery	236(58.8)	130(32.4)	106(26.4)	
	Laser	76(19.0)	44(10.9)	32(10.0)	

Key= * significant p-value

**Fig. 1:**
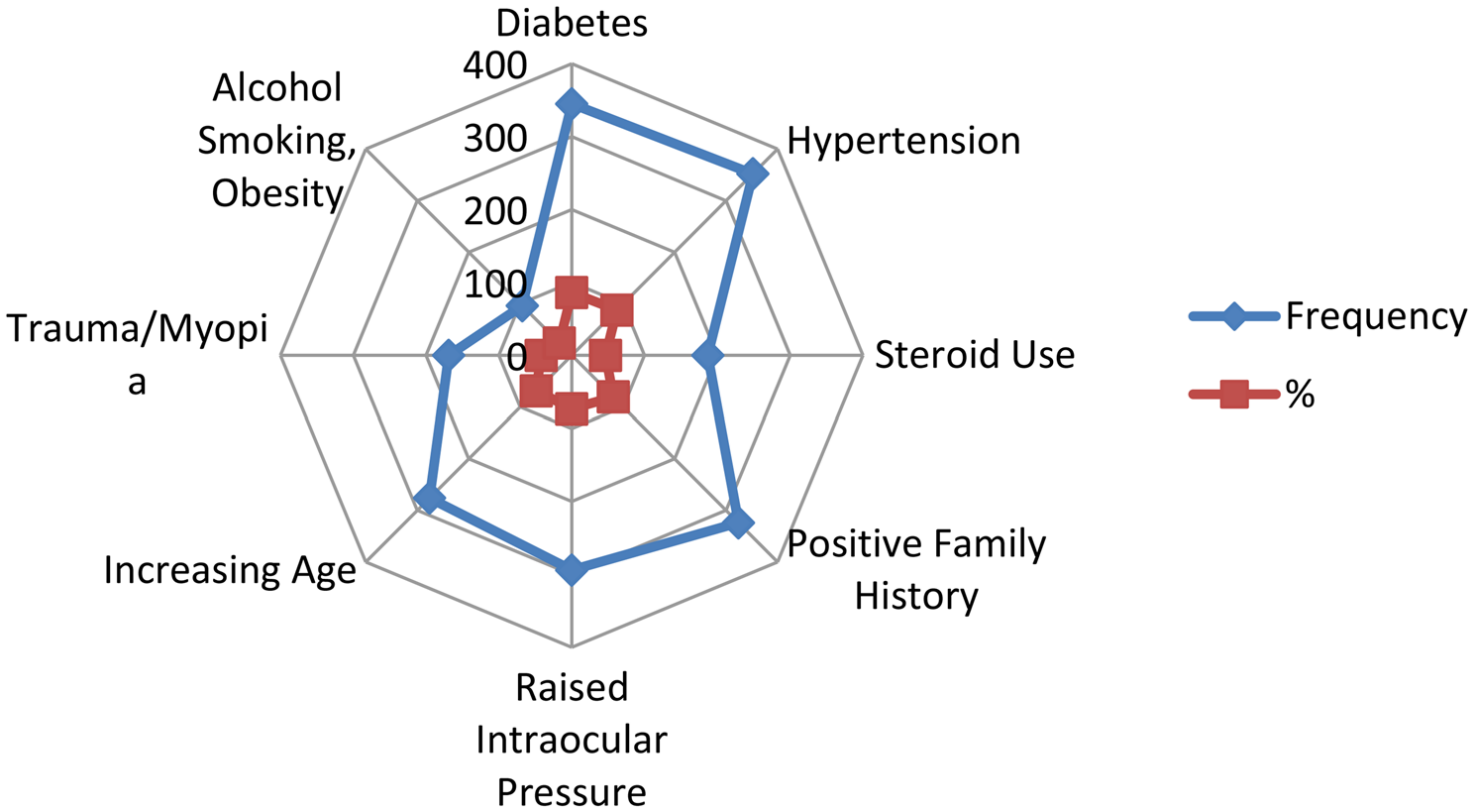
Risk factors associated with glaucoma

In Fig. 1, personnel had knowledge that hypertension 352 (87.9%), diabetes 345 (86%), positive family history of glaucoma a 324 (80.8%), raised intraocular pressure 294(73.3%), increasing age 276 (68.8%) and steroid use 187 (46.6%) were risk factors for glaucoma. Trauma and myopia were identified as risk factors by 169 (42.1%) personnel. Some workers were mistaken that alcohol consumption, smoking and obesity 96 (23.9%) caused glaucoma.

**Self-care practice**: Table 3 indicates that majority 281 (70.1%) of the personnel had never visited an ophthalmologist and only 17% had undertaken prior glaucoma screening. In the event of a glaucoma diagnosis, 324 (80.8%) workers strongly agreed they would be ready to visit the ophthalmologist or accept prescribed treatment while 57 (14.2%) did not consider it a problem to miss appointments nor miss eye drop application. About two-thirds 274(68.3%) of the responders would rather use medications than accept surgery adding that adjunctive alternatives like prayers and traditional medicines would be introduced by 236 (58.9%) responders. Medical professionals were more likely to accept prescribed protocols (p=.006) and accept surgery (p=.003) when compared to their non-medical colleagues.

**Table 3:** Attitude and self- care practice about glaucoma

When did you last visit an Ophthalmologist or Optometrist	Never (*n*,%)	Can’t Remember (*n*,%)	≥ 3 years (*n*,%)	1-2years (*n*,%)	< 1 year (*n*,%)	No Response (*n*,%)
	281(70.1)	0	52(12.9)	26(6.5)	40(10.0)	2(0.5)

Response Options	Strongly Disagree (n,%)	Disagree (n,%)	Neutral (n,%)	Agree (n,%)	Strongly Agree (n,%)	No Response (n,%)
Visit your ophthalmologist regularly and take suggested treatment consistently	0	1(0.2)	16(4.0)	57(14.2)	324(80.8)	3(0.7)
It is not an issue to miss a visit to the ophthalmologist or frequent use of eye drops	83(20.7)	208(51.9)	50(12.5)	2(0.5)	57(14.2)	1(0.2)
Will prefer getting glaucoma surgery done to avoid the hassle of putting life-long eye drops?	35(8.7)	210(52.4)	26(6.5)	104(25.9)	15(3.7)	11(2.7)
Will you promptly go ahead with surgery?	196(48.9)	63(15.7)	21(5.2)	51(12.7)	21(5.2)	49(12.2)
Try to defer surgery and continue on eye drops?	11(2.7)	6(1.5)	86(21.4)	130(32.4)	144(35.9)	24(6.0)
Take eye drops and start alternative medicine, such as traditional medication and prayers	6(1.5)	16(4.0)	37(9.2)	82(20.4)	236(58.9)	24(6.0)

Table 4 shows that 2.5% possessed good knowledge of glaucoma, while a positive attitude and good self-care practice were exhibited by 10.7% and 14.4% respectively.

**Table 4:** Grading of knowledge, attitude and self-care practice regarding glaucoma among medical and non-medical personnel

Evaluation (*n*, %)		Medical	Non-medical
Knowledge	Poor	198(49.4)	193(48.1)
	Good	8(2.0)	2(0.5)
Attitude	Negative	179(44.6)	179(44.6)
	Positive	27(6.7)	16 (4.0)
Practice	Poor	161(40.1)	182(45.4)
	Good	45(11.2)	13(3.2)
KAP	Poor	197(49.1)	194(48.4)
	Good	9(2.2)	1(0.2)

Table 5 shows the association between KAP and independent variables. The variables that were significantly associated with KAP were educational level, professional cadre, family history of glaucoma and a visit to the ophthalmologist.

Family history of glaucoma (OR 8.41, 95% CI 1.81– 39.03, P=.007) was predictive of good knowledge of glaucoma (Table 6).

**Table 5:** Association between variables and KAP of hospital personnel

Variables	Chi-square-X^2^	V-Cramer	*P* value
Sex	0.629	0.039	0.730
Age group	3.754	0.068	0.879
Marital status	5.004	0.079	0.543
Educational level	22.583	0.173	< 0.001*
Professional Cadre	35.581	0.298	< 0.001*
Personal History of glaucoma	5.519	0.084	0.479
Family History of glaucoma	26.749	0.183	0.001*
Visit to the Ophthalmologist	137.899	0.415	< 0.001*

* Significant p-value,

**Table 6:** Binary logistic regression analysis showing predictors of knowledge of glaucoma

	Coefficient B	Z	*P*	Odds Ratio	95% conf. interval
Constant	-25.89	0.003	0.99	0.00	0.00 - ∞
Gender Male	1.11	1.34	0.18	3.04	0.59–15.40
Reference Female	-	-	-	-	-
Age group(years) 18–30	-0.36	0.27	0.79	0.69	0.05–9.34
51–70	-1.10	1.02	0.30	0.33	0.04–2.76
>71	-0.23	0.00	1	0.79	0.00 - ∞
Reference 31–50	-	-	-	-	-
Marital status Married	0.82	0.75	0.45	2.28	0.26–19.74
Widowed	-19.22	0.00	1	0.00	0.00 - ∞
Reference Single	-	-	-	-	-
Educational level Secondary	-19.49	0.00	0.99	0.00	0.00 - ∞
Primary	0.76	0.00	1	2.14	0.00 - ∞
Reference Tertiary	-	-	-	-	-
Professional cadre Non-medical	-1.00	1.11	0.26	0.36	0.06–2.13
Reference Medical	-	-	-	-	-
Personal history of glaucoma; Yes	-21.27	0.00	1	0.00	0.00 - ∞
Reference No	-	-	-	-	-
Family history of glaucoma; Yes	2.13	2.72	0.007*	8.41	1.81–39.03
Reference No	-	-	-	-	-
Visit to an ophthalmologist; Yes	22.59	0.002	0.99	6512189066.2260	0.00- ∞
Reference No	-	-	-	-	-

Key: * Significant p-value, Nagelkerke R^2^ =0.443,*Confounders: Age, gender, marital status, educational level, professional cadre

Secondary level education (OR 3.35, 95% CI 1.60–7.03, *P*= .001) as shown in Table 7 was predictive of positive attitude about glaucoma.

**Table 7:** Binary logistic regression analysis showing predictors of attitude towards glaucoma

	Coefficient B	Z	*p*	Odds Ratio	95% conf. interval
Constant	-1.83	4.06	< 0.001	0.15	0.06–0.38
Gender Male	0.40	1.09	0.274	1.49	0.728– 3.06
Reference Female	-	-	-	-	-
Age group(years) 18–30	0.10	0.22	0.821	1.11	0.43–2.88
51–70	-0.77	1.21	0.225	0.46	0.13–1.61
>71	-18.61	0.00	0.999	0.00	0.00 - ∞
Reference 31–50	-	-	-	-	-
Marital Status Married	-0.12	0.30	0.762	0.87	0.38 .- 20
Widowed	-0.89	0.78	0.435	0.40	0.04–3.86
Reference: Single	--	-	-	-	-
Educational level Secondary	1.21	3.20	0.001*	3.35	1.60–7.03
Primary	-18.21	0.00	0.998	0.00	0,00- ∞
Reference Tertiary	-	-	-	-	-
Professional cadre Non-medical	-0.51	1.51	0.129	0.59	0.30–1.16
Reference Medical	-	-	-	-	-
Personal History of glaucoma Yes	-0.59	0.52	0.598	0.55	0.06–5.00
Reference No	-	-	-	-	-
Family history of glaucoma Yes	-0.62	0.98	0.324	0.53	0.15–1.86
Reference No	-	-	-	-	-
Visit to an ophthalmologist Yes	-0.36	0.89	0.368	0.69	0.30–1.54
Reference No	-	-	-	-	-

Key: * Significant p-value, Nagelkerke R^2 =^ 0.1273. *Confounders: Age, gender, marital status, educational level, Professional cadre

Relative to the medical cadre, the non-medical professional cadre was predictive of poor glaucoma practice (OR 0.22. 95% CI 0.10–0.48, *p*<.001) while a visit to the ophthalmologist (OR 19.42, 95% CI 8.78– 42.94, *p*<.001) improved glaucoma self-care practice by 19.42 times (Table 8).

**Table 8:** Binary logistic regression analysis showing predictors of glaucoma self-care practice

	Coefficient B	Z	*p*	Odds Ratio	95% conf. interval
Constant	-1.776	3.63	0.001	0.16	0.06–0.40
Gender Male	-0.56	1.25	0.208	0.56	0.23–1.36
Reference Female	-	-	-	-	-
Age group(years) 18–30	-0.49	0.96	0.336	0.60	0.21–1.68
51–70	-0.61	1.23	0.217	0.54	0.20–1.43
>71	-16.81	0.00	1	0.00	0.00- ∞
Reference 31–50	-	-	-	-	-
Marital status Married	-0.82	1.87	0.06	0.43	0.18–1.03
Widowed	-1.26	0.95	0.341	0.28	0.02–3.81
Reference Single	-	-	-	-	-
Educational level Secondary	-0.72	0.91	0.359	0.48	0.10–2.28
Primary	-18.35	0.00	0.999	0.00	0.00 - ∞
Reference Tertiary	-	-	-	-	-
Professional cadre Non medical	-1.48	3.81	< 0.001	0.22	0.10–0.48
Reference Medical	-	-	-	-	-
Personal History of glaucoma Yes	-0.15	0.12	0.9	0.85	0.08–9.12
Reference No	-	-	-	-	-
Family history of glaucoma Yes	-0.40	0.77	0.439	0.66	0.23–1.86
Reference No	-	-	-	-	-
Visit to the ophthalmologist Yes	2.96	7.33	< 0.001	19.42	8.78–42.94
Reference No	-	-	-	-	-

Key: * Significant p-value, Nagelkerke R^2 =^ 0.4274,*Confounders: Age, gender, marital status, educational level, professional cadre

## Discussions

**Knowledge of glaucoma**: Adequate knowledge (good) about glaucoma was found to be low (2.5%), findings indicate that medical personnel have a significantly better understanding of glaucoma than their non-medical counterparts (*p* < .001). This observation should be expected, given that glaucoma education is integral to the medical professionals’ training curriculum. The difference in knowledge between the cadres, underscores the urgent need for enhanced educational initiatives among all healthcare workers.

Only 26.9% and 18.6% were able to describe glaucoma as blindness and irreversible blindness respectively, which is comparatively lower than the 41% reported in rural Karnataka [13] or rates of 68%, 60%, and 48.8% from other similar studies [11, 13, 14]. In contrast to 24.7% from the index study, research from India, Ghana, and Lome [14–16] revealed that 81%, 65.9%, and 53.8%, of workers associated glaucoma with raised intraocular pressure. The association of glaucoma with optic nerve damage, peripheral field loss, and absence of symptoms early on in the diseases were equally low. These observations could be related to the fact that in the past, hospital glaucoma awareness campaigns were primarily targeted at patient and community enlightenment. In the process, personnel, particularly the non-medical workers could have been inadvertently overlooked during vital information dissemination.

The risk factors associated with POAG are varied. Though elevated intra-ocular pressure is the only modifiable risk factor, accurate knowledge of the other risk factors can help to steer glaucoma screening positively. The contribution of positive family history, increasing age, and steroid use as risk factors were similar to values from a another study [14]. Systemic hypertension (87.9%), however, was the most common risk factor in this study. The high association with glaucoma may stem from a common misconception that systemic arterial hypertension is synonymous with ocular hypertension and, by extension, glaucoma. Some personnel also incorrectly believed that alcohol consumption, smoking and obesity (23.9%) caused glaucoma. These wrong notions can be corrected when the accurate information is intentionally taught.

**Attitude**: The difference in positive attitude between medical (6.7%) and non-medical (4.0%) cadres was reflected in the medical personnel’s willingness to accept prescribed protocols (*p*=.006) and accept surgery (*p*=.003). The difference in knowledge about glaucoma between the cadres was contributory to this disparity in attitude. In the event of a glaucoma diagnosis, about two-thirds (68.6%) of the personnel expressed a preference for medical treatment over surgical intervention, which is not surprising given the fact that a combination of fear, cost and lack of accessible services affect the acceptance of surgical options by African patients [6]. Most (80.8%) of the responders indicated that they would be willing to visit an ophthalmologist and accept the recommended treatment. This finding is similar to the 77.3% compliance rate reported in another study [12]. Also, majority (91.7%) from an African study were willing to have ophthalmological follow-up review every 6 months while 65.1% opted to use eye drops for life [16]. Despite their affinity for medical management, 14.2% of the responders as opposed to just 6.7% from a previous study [11], did not view missed appointments or missed eye drop instillations as important issues. For a chronic progressive disease like glaucoma, where target intraocular pressure attainment is the goal, regular appointments and adherence to eye drop instillation are vital to the success of treatment. An attitudinal shift to actual medication compliance is imperative to the success of glaucoma regimen. This can only be achieved when workers have both knowledge and understanding about the disease.

**Self-care practice concerning glaucoma**: Female personnel outnumbered the male. A similar trend of increased female participation was noted by several researchers [9–12, 17]. This is in contrast to the findings by Amedome et al. [16], that showed the opposite trend. The female preponderance may be connected to the improving female literacy rate in Southern Nigeria [18]. The interviewed personnel work at a tertiary hospital setting, so it is not surprising that most of them had completed secondary and tertiary level education. The mean age of personnel in this study was 41.32 years, which is notably higher than the mean ages reported in other similar hospital-based [10, 14, 15] studies. Despite the high literacy among participants, being older and having easy access to an ophthalmologist, only 29.9% had ever visited one and only 17% had undergone prior glaucoma screening. This is similar to 28.6% among Ghanaian hospital workers [15] who were reported to have undergone prior ocular evaluations and 19% among Indians in Chakrabarty [14], with glaucoma screening. This observation may be linked to the paucity of qualified personnel to handle the disease. Another contributory reason may be related to the belief by respondents (8%) that blindness from glaucoma cannot be prevented with early treatment and so going through the hassle of treatment is not beneficial. The low uptake of available ophthalmology services in this study can additionally be attributed to inadequate knowledge that glaucoma blindness is irreversible and thus should be prevented. Another reason may be that personnel in a hospital setting often run hectic schedules while caring for others, neglecting their own health needs. Furthermore, because glaucoma is initially asymptomatic, many individuals may not feel the urgency to seek an ocular assessment until they begin to loose vision. Reports from an Australian [19] study found that 89% of patients attending an urban emergency department had previously undergone eye examinations suggesting that patients may have better glaucoma self-care practice than hospital workers. Consequently, if hospital personnel do not get screened as part of a regular mandatory check-up exercise, workers in the early stages of the disease may be missed.

Good self-care practice was documented in only 14.4% of the workers. This was corroborated by 58.9% being strongly open to adding adjunctive modalities to their treatment regimen, a contrast to 3.4% from an Indian study [12]. This observation suggests varied distrust of the conventional glaucoma treatment options which must be dispelled by targeted concerted re-education of hospital workers.

In our setting many persons do not volunteer for glaucoma screening until they become symptomatic or, as hospital workers, are informed about the plight of a visually impaired relative. This confirms our observation that a family history of glaucoma (*p*=.007) is predictive of a workers’ knowledge of glaucoma. A South African [20] study similarly reported that good knowledge of glaucoma translates to good self-care practice. Secondary level education (*P*= .001) was predictive of positive glaucoma attitude and a visit to the ophthalmologist did not just add to the workers knowledge but enhanced their self-care practice. The health care system and practitioners should focus not just on patients but on health workers who also need the right type of information to enable them adopt the right attitude and correct self-care practice regarding glaucoma.

## Limitation of study

The study did not identify and exclude personnel that were being managed for glaucoma prior to the research; this category of workers might have been better informed about the disease than their other colleagues. The use of interviews might have introduced recall or social desirability bias. Participants may have provided socially desirable answers rather than accurate reflection of their true attitude and practices.

## Conclusions

Medical professionals demonstrated greater understanding about glaucoma, were more likely to accept treatment protocols and consider surgical options than non-medical staff. These findings highlight the urgent need for comprehensive education and training programs for all hospital personnel. To address these gaps, several interventions are recommended. Regular glaucoma awareness seminars and training sessions should be provided for all hospital staff. Routine vision screening and glaucoma risk assessment should be included in annual occupational health checks to support early detection and management. Educational materials, such as leaflets and digital campaigns, should be developed and distributed to reinforce key messages about glaucoma risk factors, prevention, and treatment. Policies should encourage prompt referral to ophthalmology services for at-risk staff and facilitate access to affordable eye care through the National Health Insurance Scheme. These targeted educational and policy initiatives can improve knowledge, foster positive attitude, and promote better self-care practice especially among non-medical hospital workers.

## Supplementary Information

Below is the link to the electronic supplementary material.

Supplementary Material 1

## Data Availability

The datasets used and/or analyzed during the current study are available from the corresponding author on reasonable request.

## Abbreviations

KAP: Knowledge, attitude, and practice
POAG: Primary Open Angle Glaucoma
