# Supplementary material for: Knowledge, attitude, and self-care practice regarding glaucoma among hospital workers at a tertiary center in South-South Nigeria
Source: BMC Ophthalmol. 2026 Apr 17;26:192. doi: 10.1186/s12886-026-04832-7 (PMC13088400; doi:10.1186/s12886-026-04832-7)
Supplement: Supplementary file 1 — Supplementary Material 1 [file 12886_2026_4832_MOESM1_ESM.docx]

**APPENDIX 1: QUESTIONNAIRE**

**Knowledge, Attitudes, and Self-Care Practice regarding Glaucoma among Hospital Workers at a Tertiary Center in South-South Nigeria.**

**Section A: Socio-demographic characteristics.**

Kindly, tick/fill the appropriate answer to the following questions.

1. Age-------2. Sex: Male…..Female…3.Marital Status: Married-------Single------Widowed---4.Educational Level: None…………. Primary----------Secondary----------Tertiary------- 5. Department / Cadre------------
2. Do you have a relative with glaucoma? Yes…….No……..Can’t Say……..
3. Do you have glaucoma? Yes…….No……..Can’t Say……..

**Section B: Knowledge about glaucoma**

**4.** Please indicate by ticking the appropriate box (**Yes, No, Can’t Say**) about glaucoma

| **Knowledge** | Yes | No | Can’t Say |
| --- | --- | --- | --- |
| Is glaucoma associated with high pressure in the eye? |  |  |  |
| Glaucoma can cause optic nerve damage? |  |  |  |
| Can glaucoma lead to blindness? |  |  |  |
| Visual loss from glaucoma can-not be restored. |  |  |  |
| Glaucoma among Africans is painless? |  |  |  |
| Early glaucoma is asymptomatic? |  |  |  |
| Glaucoma affects side vision before central vision? |  |  |  |
| Treatment of Glaucoma is possible. |  |  |  |
| Blindness from glaucoma can be prevented by early diagnosis and treatment? |  |  |  |
| Medications /Eye drops can be used for glaucoma treatment |  |  |  |
| Surgery can be used to treat glaucoma. |  |  |  |
| Lasers are used in treating glaucoma. |  |  |  |
| Does glaucoma run in families? |  |  |  |
| Does the risk of glaucoma increase with age? |  |  |  |
| Does smoking, alcohol consumption and obesity lead to glaucoma? |  |  |  |
| Can the use of steroids increase the risk of glaucoma? |  |  |  |
| Can eye trauma and short sightedness/myopia increase the risk of glaucoma? |  |  |  |
| Are people with hypertension at higher risk of glaucoma? |  |  |  |
| Are people with diabetes at higher risk of glaucoma? |  |  |  |
| Does high eye pressure increase the risk of glaucoma? |  |  |  |

**Section C / Attitude and Self-care Practices about glaucoma**

1. **When did you last visit an ophthalmologist or an optometrist? a) < 1 year……. b) 1-2 years ……. c) ≥3 years…… d) Can’t Remember … e) Never……**
2. Please indicate (by ticking the appropriate box) if any of these statements best express your response to the questions (**where strongly disagree means a strong No and** **strongly agree is a strong Yes to the question**).

| **Attitude and Practices** | Strongly  Disagree | Disagree | Neutral | Agree | Strongly  Agree |
| --- | --- | --- | --- | --- | --- |
| If you were diagnosed with glaucoma, what would be your preferred course of treatment? | | | | | |
| Visit your ophthalmologist regularly and take suggested treatment consistently |  |  |  |  |  |
| It is not an issue to miss a visit to the ophthalmologist or frequent use of eye drops |  |  |  |  |  |
| Will prefer getting glaucoma surgery done to avoid the hassle of putting life-long eye drops? |  |  |  |  |  |
| If surgery was the only treatment option available, what would you do? | | | | | |
| Will you promptly go ahead with surgery? |  |  |  |  |  |
| Try to defer surgery and continue on eye drops? |  |  |  |  |  |
| Take eye drops and start alternative medicine, such as traditional medication and prayers |  |  |  |  |  |
